# Initiation of scutellum-derived callus is regulated by an embryo-like developmental pathway in rice

**DOI:** 10.1038/s42003-023-04835-w

**Published:** 2023-04-25

**Authors:** Fu Guo, Hua Wang, Guiwei Lian, Gui Cai, Wu Liu, Haidao Zhang, Dandan Li, Chun Zhou, Ning Han, Muyuan Zhu, Yinghua Su, Pil Joon Seo, Lin Xu, Hongwu Bian

**Affiliations:** 1 Institute of Genetic and Regenerative Biology, Key Laboratory for Cell and Gene Engineering of Zhejiang Province, College of Life Sciences, Zhejiang University, Hangzhou, 310058 China; 2 Hainan Institute, Zhejiang University, Yazhou Bay Science and Technology City, Sanya, 572025 China; 3 Yazhou Bay Seed Laboratory, Yazhou Bay Science and Technology City, Yazhou District, Sanya, 572025 China; 4 National Key Laboratory of Plant Molecular Genetics, CAS Center for Excellence in Molecular Plant Sciences, Institute of Plant Physiology and Ecology, Chinese Academy of Sciences, 300 Fenglin Road, Shanghai, 200032 China; 5 University of the Chinese Academy of Sciences, 19A Yuquan Road, Beijing, 100049 China; 6 State Key Laboratory of Crop Biology, Shandong Key Laboratory of Crop Biology, College of Life Sciences, Shandong Agricultural University, Taian, Shandong 271018 China; 7 Department of Chemistry, Seoul National University, Seoul, 08826 Korea; 8 Plant Genomics and Breeding Institute, Seoul National University, Seoul, 08826 Korea; 9 Present Address: Institute of Cell Biology, School of Biological Sciences, The University of Edinburgh, Edinburgh, UK

**Keywords:** Plant regeneration, Plant stem cell

## Abstract

In rice (*Oryza sativa*) tissue culture, callus can be induced from the scutellum in embryo or from the vasculature of non-embryonic organs such as leaves, nodes, or roots. Here we show that the auxin signaling pathway triggers cell division in the epidermis of the scutellum to form an embryo-like structure, which leads to callus formation. Our transcriptome data show that embryo-, stem cell-, and auxin-related genes are upregulated during scutellum-derived callus initiation. Among those genes, the embryo-specific gene *OsLEC1* is activated by auxin and involved in scutellum-derived callus initiation. However, *OsLEC1* is not required for vasculature-derived callus initiation from roots. In addition, *OsIAA11* and *OsCRL1*, which are involved in root development, are required for vasculature-derived callus formation but not for scutellum-derived callus formation. Overall, our data indicate that scutellum-derived callus initiation is regulated by an embryo-like development program, and this is different from vasculature-derived callus initiation which borrows a root development program.

## Abstract (web summary)

Molecular and cellular characterization of callus formed from the rice scutellum reveals involvement of an embryo-like developmental program, in contrast to callus formed from the vasculature, which is regulated by genes involved in root organogenesis.

## Introduction

Callus is a group of fast-dividing parenchyma cells, and many types of callus could be formed in tissue culture or upon wounding^1–3^. Studies in the dicot model plant *Arabidopsis thaliana* and the monocot model plant rice (*Oryza sativa*) indicated that callus could be induced from different regeneration-competent cells of explants in tissue culture. For example, the vascular adult stem cells may serve as regeneration-competent cells to initiate callus (hereafter described as vasculature-derived callus) in *Arabidopsis*^4–7^ and rice^7^, and the epidermal cells of the rice scutellum, which is the rice cotyledon in embryo, can also act as regeneration-competent cells responsible for the initiation of callus^8,9^ (hereafter described as scutellum-derived callus).

Initiation of the vasculature-derived callus borrows the root developmental pathway^4–7,10–16^. In *Arabidopsis*, lateral or adventitious roots are initiated from adult stem cells in vasculature, e.g., xylem pole pericycle cells in roots or procambium and some vascular parenchyma cells in leaves, and those adult stem cells are responsible for the initiation of vasculature-derived callus in tissue culture^4–7,17^. In rice, vasculature-derived callus can be initiated from outer bundle sheath cells in the immature region of leaves or from phloem-pole pericycle cells in roots^7^, and the phloem-pole pericycle cells also initiate lateral roots in rice root system formation.

The cellular structure of vasculature-derived callus resembles the root primordium (RP) and root apical meristem (RAM)^6,7,13^. Studies in *Arabidopsis* revealed that many genes related to RP/RAM are highly expressed in vascular-derived callus, including the WUSCHEL-RELATED HOMEOBOX (WOX) transcription factor family genes *AtWOX5* and *AtWOX7* (*AtWOX5*/*7*) and the APETALA2 (AP2)-like transcription factor family genes *PLETHORA1* and *2* (*AtPLT1*/*2*) and *AtPLT3*/*5*/*7*^6,13,14,18,19^. In rice, *OsWOX5* is also highly expressed in vasculature-derived callus^7^.

In rice tissue culture, scutellum-derived callus is initiated from the epidermal cells of the scutellum^8,9^. Genetic, transcriptome, and epigenome analyses have revealed many gene networks in the formation of scutellum-derived callus in rice^9,20–23^. However, our knowledge on scutellum-derived callus formation in rice is still limited, and the developmental and molecular strategies adopted in scutellum-derived callus formation in comparison with vasculature-derived callus formation need to be explored. In this study, we propose that scutellum-derived callus initiation might borrow the embryo development program in rice.

## Results

### Auxin signaling pathway mediates scutellum-derived callus initiation

**Fig. 1:**
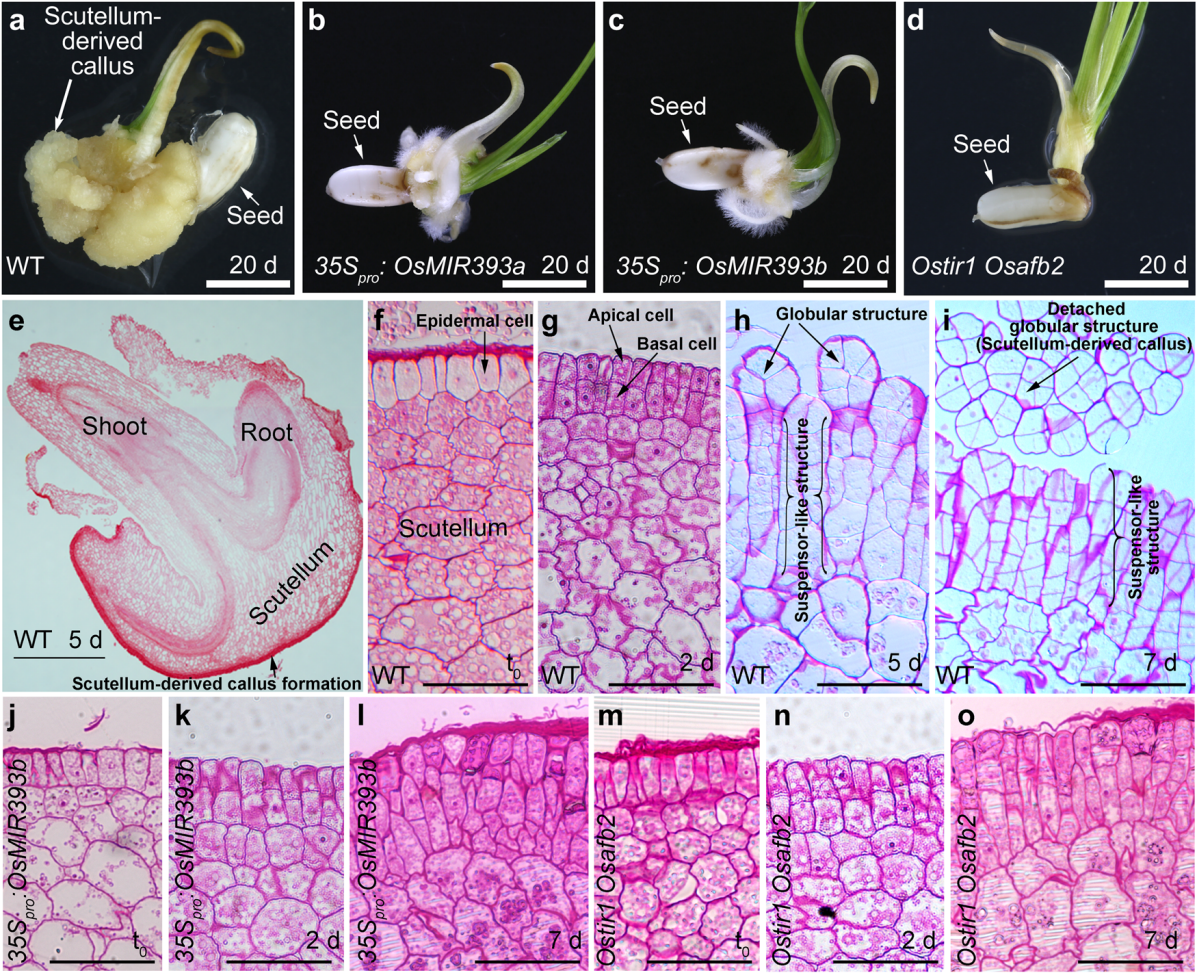
Developmental framework of auxin-mediated callus formation from scutellum in rice. **a**–**d** Tissue culture of rice mature seeds from wild-type (WT) (**a**), *35S*_*pro*_*:OsMIR393a* (**b**), *35S*_*pro*_*:OsMIR393b* (**c**), and *Ostir1 Osafb2* (**d**) on CIM for 20 d. We tested more than 90 wild-type seeds, and all of them formed scutellum-derived callus. We tested more than 30 seeds from *35S*_*pro*_*:OsMIR393a*, *35S*_*pro*_*:OsMIR393b*, or *Ostir1 Osafb2*, and none of them formed scutellum-derived callus. **e**–**i** Sections showing callus formation from scutellum (**e**) of wild-type rice seeds on CIM at t_0_ (**f**), 2 d (**g**), 5 d (**e**, **h**), and 7 d (**i**). See Supplementary Fig. 2 for details. **j**–**o** Sections showing cell division from scutellum of *35S*_*pro*_*:OsMIR393b* (**j**–**l**) and *Ostir1 Osafb2* (**m**–**o**) seeds on CIM at t_0_ (**j**, **m**), 2 d (**k**, **n**), and 7 d (**l**, **o**). Scale bars, 5 mm (**a**–**d**), 500 μm (**e**), 50 μm (**f**–**o**).

To study the role of auxin in scutellum-derived callus formation, we cultured mature seeds of wild-type rice on callus-inducing medium (CIM) with a high level of auxin. Many calli were visible to the naked eye at 20 days of culture (Fig. 1a). Overexpression of *OsMicroRNA393a* (*35S*_*pro*_*:OsMIR393a*) and *OsMIR393b* (*35S*_*pro*_*:OsMIR393b*), which can target and degrade the mRNA of the auxin receptor genes *TRANSPORT INHIBITOR RESPONSE1* (*OsTIR1*) and *AUXIN SIGNALING F BOX PROTEIN2* (*OsAFB2*)^24^, resulted in the loss of scutellum-derived callus formation on CIM^25^ (Fig. 1b, c; Supplementary Fig. 1). Similarly, double mutations in *OsTIR1* and *OsAFB2*^24^ also led to defects in scutellum-derived callus formation (Fig. 1d; Supplementary Fig. 1). Therefore, auxin could be the key hormone that triggers scutellum-derived callus formation in rice.

To study the cellular origin and developmental progress during scutellum-derived callus initiation, we analyzed sections of wild-type mature seeds on CIM. Callus was initiated from the epidermal cells of the scutellum (Fig. 1e)^8^. The epidermal cell started to divide at 2 days of culture on CIM, resulting in an apical cell and a basal cell (Fig. 1f, g; Supplementary Fig. 2). The apical and basal cells continued to divide and form an embryo-like structure, in which the apical cell had formed a globular structure and the basal cell had formed a suspensor-like structure at 5 days of culture on CIM (Fig. 1h; Supplementary Fig. 2). Cell division continued in the globular structure, which gradually detached from the suspensor-like structure (Fig. 1i; Supplementary Fig. 2). The detached globular structure continued cell division to form the scutellum-derived callus (Fig. 1i; Supplementary Fig. 2).

Cell division occurred in the epidermal cells of *35S*_*pro*_*:OsMIR393b* and the *Ostir1 Osafb2* double mutant (Fig. 1j–o), but the dividing cells could not further develop to form the embryo-like structure (Fig. 1l, o). Therefore, no scutellum-derived callus could be formed and detached from the scutellum.

**Fig. 2:**
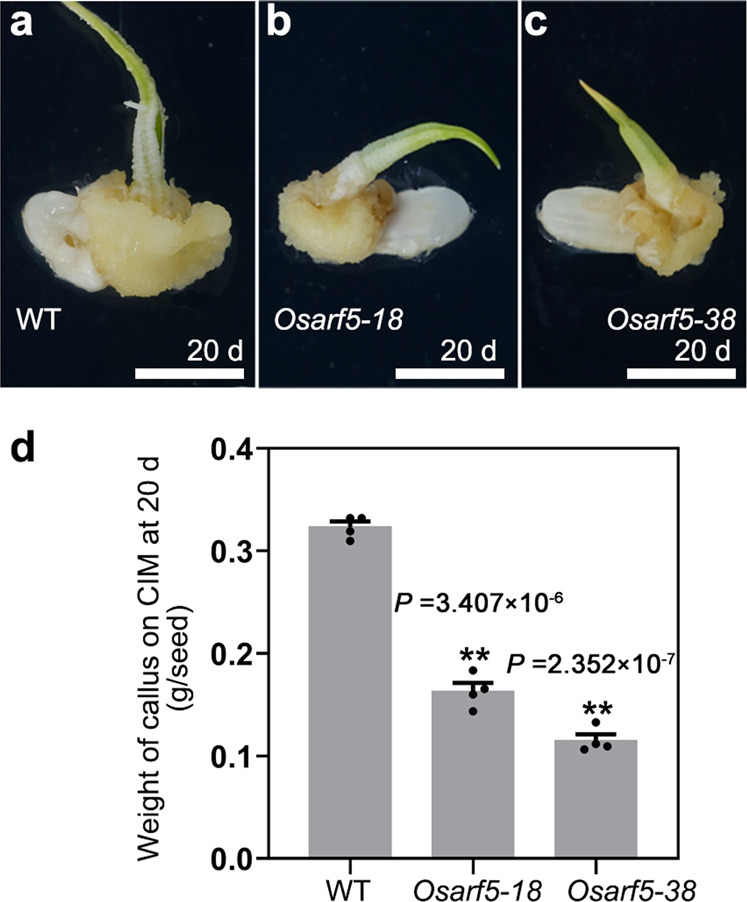
OsARF5 is involved in scutellum-derived callus formation. **a**–**c** Phenotype of callus formation from the scutella of wild-type (**a**) *Osarf5-18* (**b**) and *Osarf5-38* (**c**) on CIM at 20 d. **d** Statistical analysis of scutellum-derived callus formation in wild-type, *Osarf5-18*, and *Osarf5-38* on CIM at 20 d. The individual values are indicated by dots. The data are presented as mean values ± s.e.m. from four biological replicates (10 calli in each replicate). **P < 0.01 in two-sided Student’s *t*-test. Scale bars, 5 mm (**a**–**c**).

To confirm that the auxin signaling pathway is essential for rice scutellum-derived callus formation on CIM, we created the mutations in *AUXIN RESPONSE FACTOR5* (*OsARF5*) by CRISPR/Cas9 (Supplementary Fig. 3). The *Osarf5* mutants showed partially defective formation of scutellum-derived callus (Fig. 2).

Analysis of the β-glucuronidase (GUS) marker line *DR5*_*pro*_*:GUS* showed that the auxin response was not activated in the scutellum before tissue culture (Supplementary Fig. 4a, b, f, g), and was highly active in the dividing cells in the epidermis of the scutellum (Supplementary Fig. 4c–e, h–j) and in the detached globular structure (i.e. scutellum-derived callus) (Supplementary Fig. 4e). Analysis of *OsTIR1*_*pro*_*:GUS* showed that *OsTIR1* was expressed in the scutellum at all stages in tissue culture (Supplementary Fig. 4k–t).

Overall, these data indicate that the auxin signaling pathway is required for the proper cell division of the scutellum epidermal cell to initiate an embryo-like structure, which leads to the formation of scutellum-derived callus.

### Initiation of scutellum-derived and vasculature-derived calli adopts different molecular strategies

**Fig. 3:**
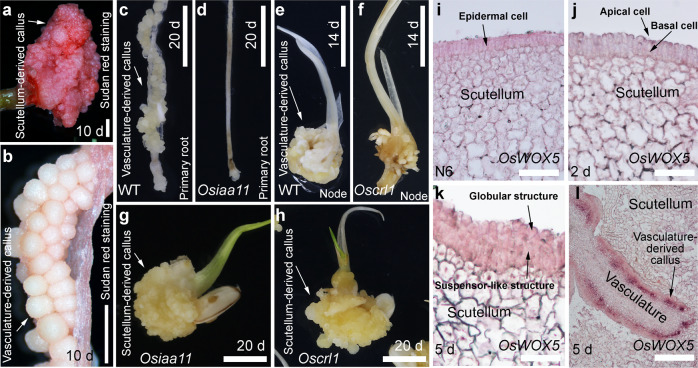
Analysis of scutellum-derived and vasculature-derived calli in rice. **a**, **b** Sudan red staining of scutellum-derived callus from mature seeds (**a**) or vasculature-derived callus from root (**b**) on CIM at 10 d. **c**, **d** Primary roots of wild-type (**c**) and *Osiaa11* (**d**) tissue-cultured on CIM for 20 d. **e**, **f** Nodes of wild-type (**e**) and *Oscrl1* (**f**) tissue-cultured on CIM for 14 d. **g**, **h** Seeds of *Osiaa11* (**g**) and *Oscrl1* (**h**) tissue-cultured on CIM for 20 d. **i**–**l** In situ hybridization using anti-*OsWOX5* probe in scutellum (**i**–**k**) and vasculature (**l**) of wild-type embryos in mature seeds at 2 d (**j**) or 5 d (**k**, **l**). Seeds cultured on N6 medium without hormone treatment for 2 d served as the control (**i**), because t_0_-seeds were dry and unsuitable for in situ hybridization. Some vasculatures in the embryo can initiate vasculature-derived callus with *OsWOX5* expression (**l**). Scale bars, 1 mm (**a**, **b**), 5 mm (**c**–**h**), 50 μm (**i**–**k**), 500 μm (**l**).

We next tested whether scutellum-derived callus formation and vasculature-derived callus formation share similar genetic pathways in rice. Scutellum-derived callus showed positive staining of Sudan red (Fig. 3a), while vasculature-derived callus formed from the root explant did not (Fig. 3b), indicating that scutellum-derived callus but not vasculature-derived callus has the embryo-like cell identity that is abundant in lipids. The *indole-3-acetic acid inducible11* (*Osiaa11*) mutant, which is defective in lateral root formation^26^, showed defective vasculature-derived callus formation from the primary root on CIM (Fig. 3c, d). The *crown rootless1* (*Oscrl1*) mutant, which is defective in adventitious root formation from the node^27^, showed defective vasculature-derived callus formation from the node on CIM (Fig. 3e, f). However, normal scutellum-derived callus was able to be produced from the mature seeds of both *Osiaa11* and *Oscrl1* culture on CIM (Fig. 3g, h). In addition, the vasculature-derived callus marker gene *OsWOX5*^7^, which is a root apical stem cell niche-related gene^28,29^, was not highly induced during the division of the scutellum epidermal cells in scutellum-derived callus initiation (Fig. 3i–k), while its expression was induced in the vasculature of the embryo to form vasculature-derived callus (Fig. 3l). These data indicate that rice vasculature-derived callus adopts *OsIAA11*/*OsCRL1*/*OsWOX5*-mediated root development program, while scutellum-derived callus does not.

### Transcriptome framework of scutellum-derived callus formation

We carried out RNA-seq analysis using mature embryos of wild-type and *35S*_*pro*_*:OsMIR393b* at 2, 5 and 7 days of culture on CIM, compared with the control (Supplementary Fig. 5). The analysis of wild-type transcriptome might reveal the genes involved in scutellum-derived callus formation, and the transcriptome comparison of the wild-type and *35S*_*pro*_*:OsMIR393b* could indicate the genes regulated by the auxin signaling pathway during scutellum-derived callus formation.

**Fig. 4:**
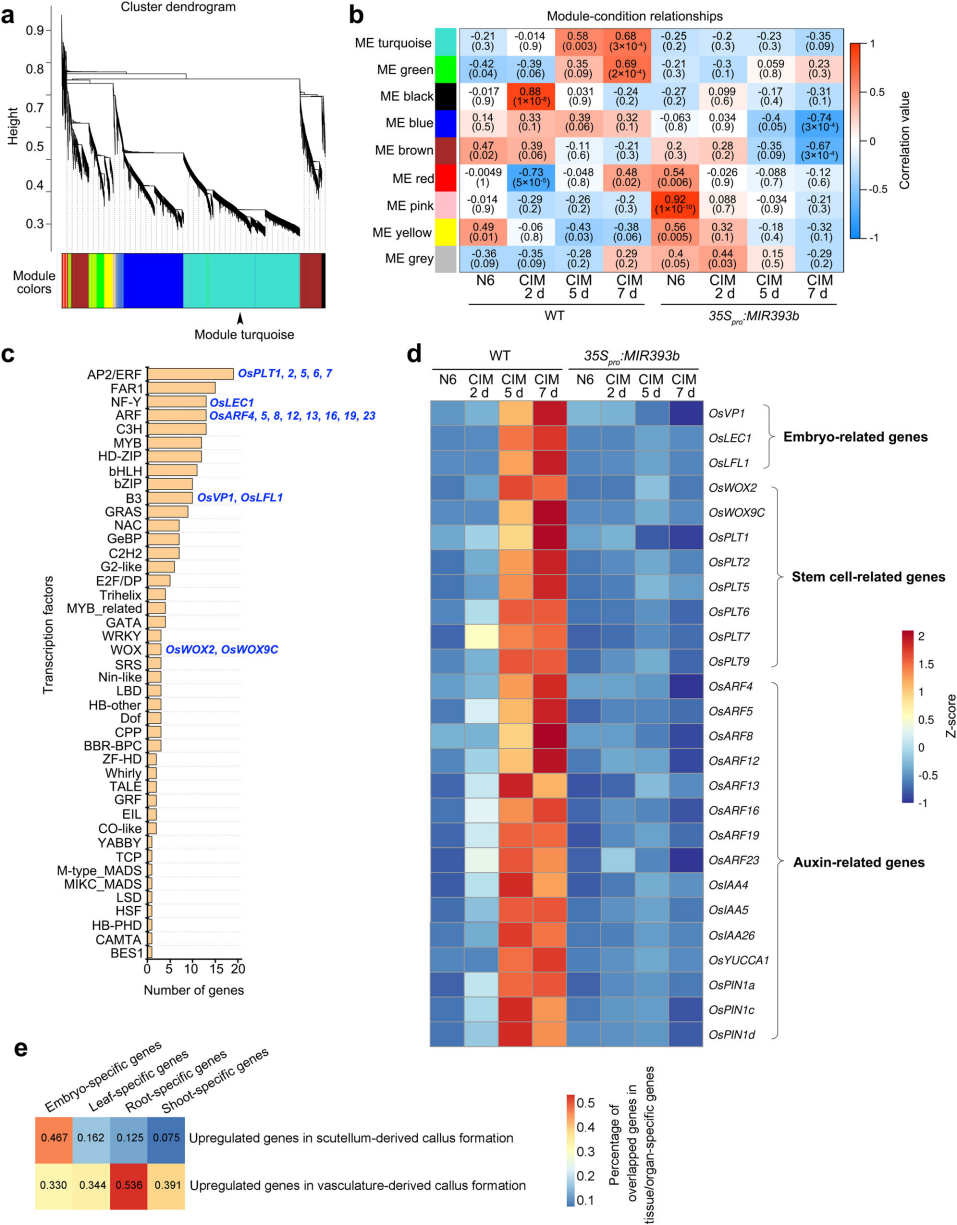
RNA-seq analysis reveals auxin-activated genes in rice scutellum-derived callus formation. **a** RNA-seq and WGCNA analysis of scutella from wild-type and *35S*_*pro*_*:OsMIR393b* at 2, 5, and 7 d cultured on CIM. Wild-type and *35S*_*pro*_*:OsMIR393b* scutella cultured on N6 medium for 2 d without hormone treatment served as the control, because t_0_-seeds were dry and unsuitable for analysis. Genes with high correlations are grouped into a module, and different modules are indicated by different colors. **b** Correlation analyses between module eigengene (ME) values (ME turquoise, ME green, ME black, ME blue, ME brown, ME red, ME pink, ME yellow, ME gray) and each culture conditions (N6, CIM 2 days, CIM 5 days, CIM 7 days). ME value is the representative of the expression profile of all genes in the corresponding module. Numbers indicate the correlation coefficient, and the numbers in brackets indicate *P* values for the correlation. The ME turquoise showed a high correlation with scutellum-derived callus formation in the wild-type background but not in the *35S*_*pro*_*:OsMIR393b* background. Therefore, the genes in module turquoise were used for further analysis in (**c**, **d**). **c** Bar chart analysis of the frequency of transcription factor families. **d** Relative expression levels of embryo-, stem cell-, and auxin-related genes. **e** Overlapped genes of upregulated genes in vasculature- or scutellum-derived callus formation and embryo-, leaf-, root-, or shoot-specific expression genes are identified. The percentage of overlapped gene numbers in tissue/organ-specific genes are shown by heatmap.

Weighted Correlation Network Analysis (WGCNA)^30^ was performed and nine modules were identified (Fig. 4a, b, Supplementary Data 1). The module eigengene (ME) turquoise showed a high correlation with scutellum-derived callus formation in the wild-type background but not in the *35S*_*pro*_*:OsMIR393b* background (Fig. 4b), indicating the module turquoise might be controlled by the auxin signaling pathway during scutellum-derived callus formation. Gene Ontology (GO) enrichment (Supplementary Fig. 6) analyses showed that genes related to cell division as well as many other biological processes were enriched in the module turquoise. We identified 229 transcription factors in the top 30% hub genes of the module turquoise, including some key genes related to embryo regulation [e.g. the *Arabidopsis LEAFY COTYLEDON1* (*AtLEC1*) homolog *OsLEC1*, the *Arabidopsis ABSCISIC ACID INSENSITIVE3* (*AtABI3*) homolog *VIVIPAROUS1* (*OsVP1*), and *O. sativa LEC2 and FUSCA3 Like1* (*OsLFL1*)], stem cell regulation (*OsWOX2*/*9c* and *OsPLT*s), and auxin production, signaling, and transport [*OsYUCCA1*, *OsIAA*s, *OsARF*s, and *OsPIN-FORMED*s (*OsPIN*s)] (Fig. 4c, d). The RNA-seq data showed that expression levels of these key genes were gradually upregulated during scutellum-derived callus formation on CIM in the wild-type background but this upregulation was severely impaired in the *35S*_*pro*_*:OsMIR393b* background (Fig. 4d). Reverse transcription-polymerase chain reaction (RT-PCR) analyses confirmed that *OsLEC1*, *OsWOX2*, and *OsWOX9C* were upregulated during scutellum-derived callus formation from wild-type rice seeds on CIM (Supplementary Fig. 7).

We further analyzed transcriptome correlation of callus and rice tissues or organs. We collected RNA-seq data of four rice tissues or organs (i.e. embryo^31^, leaf^32^, root^33^, and shoot^33^), and identified tissue/organ-specific expression genes. By comparison of upregulated genes from the RNA-seq data of vasculature-derived callus^7^ and scutellum-derived callus (data from this study) with those tissue/organ-specific expression genes, we found that root-specific genes were highly enriched in the upregulated genes during vasculature-derived callus formation, whereas embryo-specific genes were highly enriched in the upregulated genes during scutellum-derived callus formation (Fig. 4e).

### *OsLEC1* is required for scutellum-derived callus initiation

**Fig. 5:**
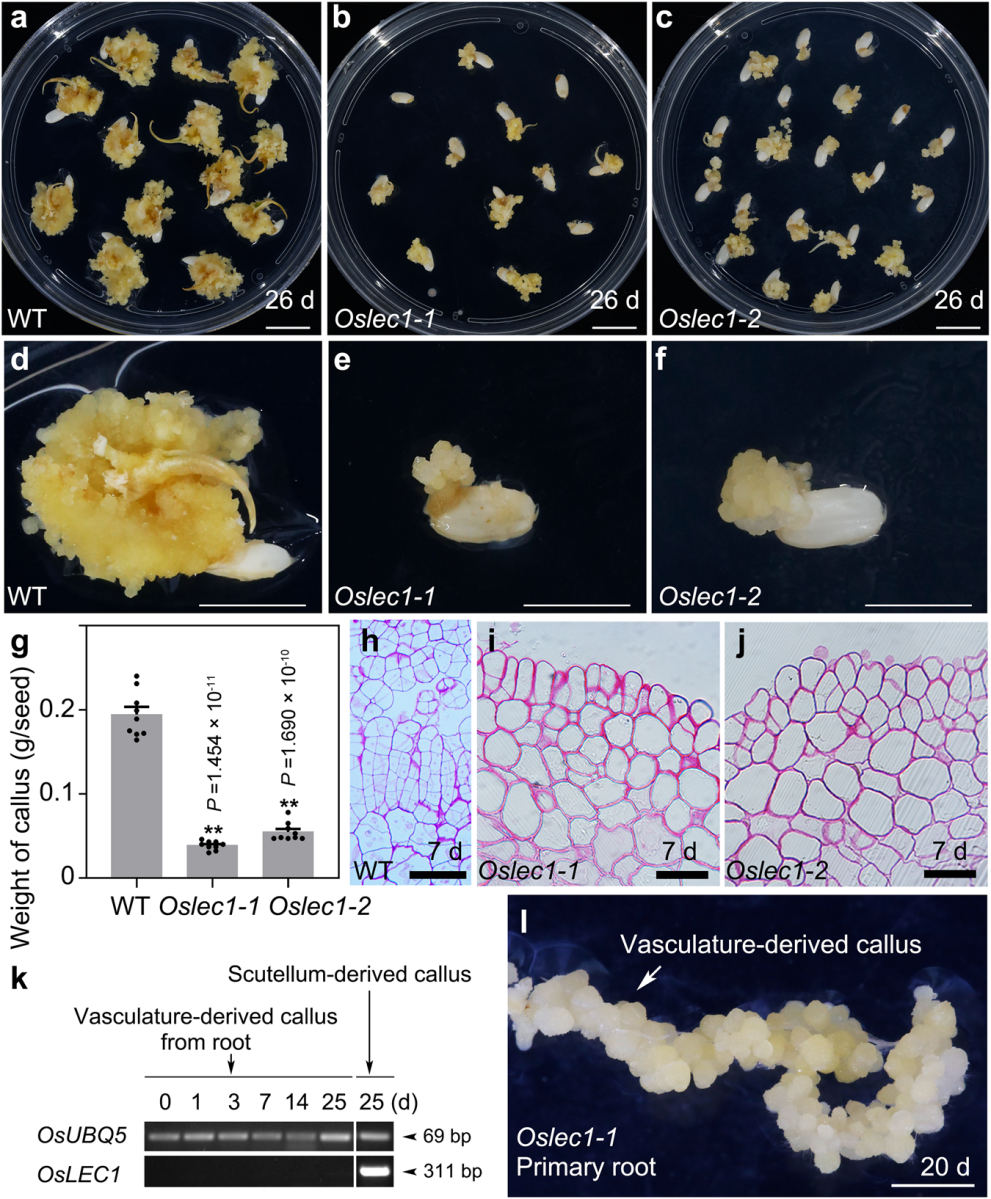
*OsLEC1* is involved in scutellum-derived callus formation. **a**–**f** Rice seeds from wild-type (**a**, **d**), *Oslec1-1* (**b**, **e**), and *Oslec1-2* (**c**, **f**) tissue-cultured on CIM for 26 d. **g** Statistical analysis of scutellum-derived callus formation in wild-type, *Oslec1-1*, and *Oslec1-2* on CIM at 16 d. Error bars show SEM with nine biological replicates (10 calli in each replicate). The individual values are indicated by dots. **P < 0.01 in two-sided Student’s *t*-test compared with the wild-type control. **h**–**j** Sections showing cell division of scutellum epidermis from wild-type (**h**), *Oslec1-1* (**i**) and *Oslec1-2* (**j**) seeds on CIM at 7 d. **k** RT-PCR analysis of *OsLEC1* expression pattern in vasculature-derived callus and scutellum-derived callus (33 cycles). *OsUBQ5* serves as the control (30 cycles). Numbers indicate the days cultured on CIM. The bands of *OsUBQ5* and *OsLEC1* are derived from different gels. The bands from vasculature-derived callus and scutellum-derived callus were separately collected from the same gel, and the experiments were performed under the same conditions. The predicted band sizes were indicated. Two biological repeats were analyzed and showed the same result. **l** Primary roots of *Oslec1-1* tissue-cultured on CIM for 20 d, showing vasculature-derived callus formation. See Fig. 3c for the wild-type control. Scale bars, 1 cm (**a**–**c**), 5 mm (**d**–**f**), 50 μm (**h**–**j**), 1 mm (**l**).

To confirm the auxin-activated genes detected from the RNA-seq data are required for scutellum-derived callus formation in rice, we selected the embryo-specific gene *OsLEC1* for further analysis. The *Oslec1* mutants^31^ showed partially defective scutellum-derived callus formation (Fig. 5a–g). Successive cell division of the scutellum epidermis was lost and the embryo-like structure could not be formed in the *Oslec1* mutants in tissue culture (Fig. 5h–j). Overexpression of *OsLEC1* also resulted in partially defective callus formation^31^, suggesting that the proper level of *OsLEC1* expression might be important for scutellum-derived callus formation. The *OsLEC1*_*pro*_*:GUS* marker line^31^ indicated that the *OsLEC1* promoter might be highly activated in the scutellum-derived callus (Supplementary Fig. 8a, b). The RT-PCR result showed that *OsLEC1* was expressed during scutellum-derived callus formation but was not expressed during vasculature-derived callus formation from the primary root (Fig. 5k). In addition, the *Oslec1* primary root could produce vasculature-derived callus (Fig. 5l). The study in *AtLEC* genes also showed that *AtLEC*s are not required for organ regeneration from vasculature-derived callus in *Arabidopsis*^34^. qRT-PCR analysis revealed that the *OsLEC1* expression level is significantly reduced in the *Osarf5* mutants compared with the wild-type when mature seeds were cultured on CIM (Supplementary Fig. 8c), indicating that *OsLEC1* might be upregulated by the *OsARF5*-mediated auxin signaling pathway^35^. Together, these data indicate that *OsLEC1* is involved in auxin-mediated scutellum-derived callus formation from the rice scutellum but is not required for vasculature-derived callus formation.

## Discussion

**Fig. 6:**
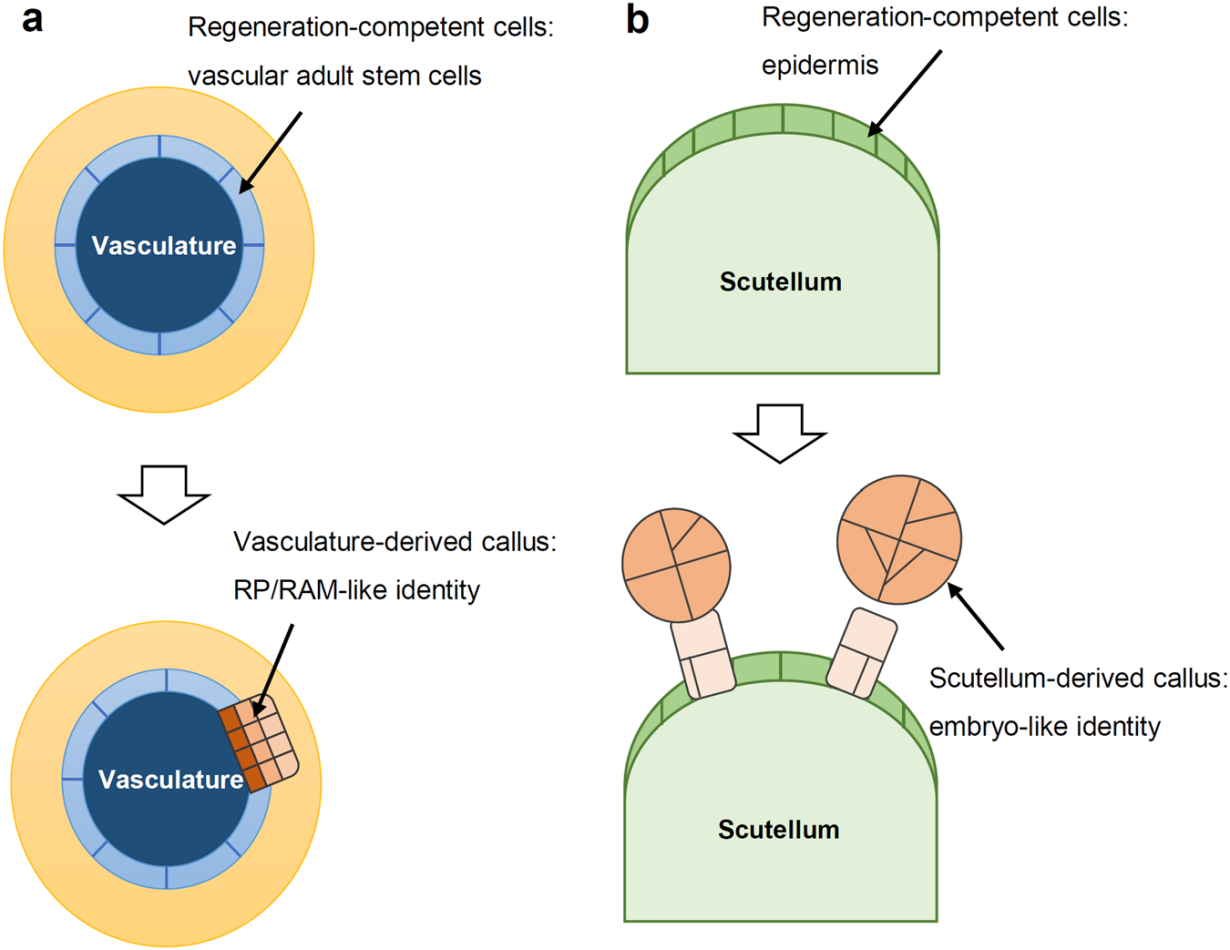
Model of callus initiation in rice. **a**, **b** Vasculature-derived callus initiation adopts a root developmental program (**a**), and scutellum-derived callus initiation might adopt a embryo developmental program (**b**).

Auxin is the key hormone that triggers initiation of both scutellum-derived callus and vasculature-derived callus. However, there are some differences between the two types of calli (see the model in Fig. 6).

First, the two types of calli initiate from different regeneration-competent cells. In *Arabidopsis*, vasculature-derived callus initiates from the xylem-pole pericycle cells in roots^4–6^ or procambium and some vascular parenchyma cells in leaf^7,17^. In rice, vasculature-derived callus initiates from phloem-pole pericycle cells in roots or outer bundle sheath cells in the immature region of leaves^7^. In rice, the epidermal cells of the scutellum may serve as the regeneration-competent cells to initiate scutellum-derived callus^8^. Overall, vascular adult stem cells are responsible for vasculature-derived callus initiation, while epidermis of embryo could be the primary source to initiate scutellum-derived callus.

Second, the two types of calli show different developmental morphologies. The tissue structure of vasculature-derived callus resembles RP/RAM^6,10,11^ and comprises three cell layers, i.e. the outer cell layer similar to epidermis and lateral root cap, the middle cell layer with quiescent center-like identity, and the inner cell layer similar to vascular initials in the RAM^13,19^. Initiation of scutellum-derived callus resembles embryogenesis. The epidermis of scutellum divides and developments into an embryo-like morphology with a globular structure and a suspensor-like structure.

Third, scutellum-derived and vasculature-derived calli may require some different molecular and developmental programs for their initiation. In *Arabidopsis*, vasculature-derived callus initiation requires root-organogenesis-related genes, e.g. *AtLBD16*, *AtWOX5*, *AtPLTs*, and *AtIAA14*^10,12–14,16,18,36^. In rice, *OsCRL1* (the *AtLBD16* homologue), *OsWOX5* (the *AtWOX5* homologue), and *OsIAA11* (the *AtIAA14* homologue) are also required for vasculature-derived callus formation^7,16^. In contrast, initiation of scutellum-derived callus does not require some of the root-organogenesis-related genes in rice (e.g. *OsCRL1* and *OsIAA11*). Instead, genes involved in embryo development are required in scutellum-derived callus formation in rice (e.g. *OsLEC1*). Overall, vasculature-derived callus initiation borrows root development program, whereas scutellum-derived callus initiation might borrow the embryo development program.

The developmental similarities of scutellum-derived callus and somatic embryogenesis need to be further explored. Somatic embryogenesis might also borrow some pathways from embryo development. In *Arabidopsis*, somatic embryogenesis can be initiated from the epidermis in the junction of the shoot apical meristem and the cotyledon in embryo controlled by a high level of auxin^37^. In *Arabidopsis*, somatic embryogenesis essentially involves two groups of genes^38^. The first group consists of stem cell-related genes, such as *AtWUS*^39–41^, *AtWOX2*^37^, *BABYBOOM* (*AtBBM*, also known as *AtPLT4*)^42,43^, and *EMBRYOMAKER* (*AtEMK*, also known as *AtPLT5*)^44^. The second group consists of embryo-related genes, including *AtLEC1* encoding the HAP3 subunit of the CCAAT-binding transcription factor^45–47^, the B3-domain genes *AtLEC2*^48–51^, *FUSCA3* (*AtFUS3*)^51–53^, *AtABI3*^47^, and the MADS-box transcription factor gene *AGAMOUS-LIKE15* (*AtAGL15*)^54–57^. Some homologues of these genes were upregulated and involved in scutellum-derived callus in rice. It will be interesting to compare the initiation processes of scutellum-derived callus and somatic embryogenesis in the future.

There are many types of calli induced in response to wounding or in tissue culture^1–3,58,59^. It will be interesting to identify more types of cells that can serve as regeneration-competent cells to initiate callus, to analyze how auxin can induce different types of calli from different types of explants, to test whether/how different types of calli can undergo organ regeneration and/or somatic embryogenesis, to study whether/how different types of calli can be converted to each other, and to see whether different types of calli share some common molecular pathways (e.g. the stem cell-related pathway) during their initiation in the future. For example, scutellum-derived and vasculature-derived calli might share some common regulatory pathways^20,21^, and *PLT* genes were upregulated during initiation of the two types of calli (Fig. 4c, d)^6,13,14,19,21^. The answers to these questions will improve our understanding of callus formation in the future.

## Methods

### Plant materials

*Oryza sativa* L. *ssp*. Japonica ‘Nipponbare’, ‘Taichung 65’, and Indica ‘Kasalath’ were used as the wild-type lines. The *35S*_*pro*_*:MIR393a*, *35S*_*pro*_*:MIR393b*, *Ostir1 Osafb2*, *Oslec1-1*, *Oslec1-2*, *DR5*_*pro*_*:GUS*, and *OsTIR1*_*pro*_*:GUS* transgenic plants (Nipponbare background) as well as *Oscrl1* (Taichung 65 background) and *Osiaa11* (Kasalath background) have been described in previous studies^23,27,31,60,61^. To generate *OsARF5* mutants, *OsARF5*-specific sequences (5′-AGTATCTGGGTCTGCCTGAT-3′ and 5′-GGAATATCTTCTCTGCGGCA-3′) were used as the targets for Cas9 to mutate *OsARF5* by CRISPR/Cas9^62^.

### Tissue culture

For time-lapse histological observations of callus induction from the scutellum, sterile seeds of wild type or mutants were placed on N6 medium (0.5 g l^–1^ MES, 3% w/v sucrose, and 0.4% w/v phytagel, pH 5.8) or CIM medium (N6 basal medium supplemented with 10 μM 2, 4-dichlorophenoxyacetic acid, pH 5.8) and cultured in a growth chamber under a 16-h light, 28 °C/8-h dark, 24 °C cycle^16^. The scutellum was detached as described previously^23^ and fixed at 4 °C in 2.5% v/v glutaraldehyde in phosphate buffer (0.1 M, pH 7.0). Scutella collected from dry seeds and fixed directly in 2.5% v/v glutaraldehyde served as the t_0_ control.

For vasculature-derived callus induction from the primary root, seeds of Kathalath and the *Osiaa11* mutant were sterilized, soaked in water overnight at 37 °C, sown on Murashige and Skoog (MS) medium^63^, and then grown vertically in a growth chamber for 3 days under a 16-h light, 28 °C/8-h dark, 26 °C cycle. The primary roots of 3-d-old seedlings were cut and cultured on N6 medium or CIM.

For vasculature-derived callus induction from the stem base (the node), seeds of Taichung 65 and the *Oscrl1* mutant were sterilized and then cultured on MS medium for 3 days under a 16-h light, 28 °C/8-h dark, 26 °C cycle. The 3-d-old seedings were cut and cultured on CIM.

Three independent repeats were analyzed to confirm the results of phenotype analysis.

### In situ hybridization and Sudan red staining

In situ hybridization was performed as described previously using the probe for *OsWOX5*^7^. Sudan red staining was performed according to the published protocol^64^.

### Histochemical detection of GUS activity

For β-glucuronidase (GUS) staining, tissues were incubated in X-Gluc solution overnight at 37 °C as described previously^61^, and then fixed in pre-chilled 2.5% v/v glutaraldehyde in phosphate buffer (0.1 M, pH 7.0) overnight before being photographed and/or sectioned.

### Semi-thin sectioning

Semi-thin sectioning was performed as described previously^65^. Briefly, tissues were fixed overnight at 4 °C in 2.5% v/v glutaraldehyde in phosphate buffer (0.1 M, pH 7.0), and then washed three times in phosphate buffer (0.1 M, pH 7.0) for 15 min at each step. The samples were dehydrated through an ethanol series (30, 50, 70, 80, 90, 95, and 100% ethanol, 15 min each), then immersed in absolute acetone for 20 min. After substitution with a 1:1 mixture of absolute acetone and Spurr’s resin mixture for 1 h, the tissues were transferred to a 1:3 mixture of absolute acetone and Spurr’s resin mixture for 3 h, and then embedded in pure Spurr’s resin mixture overnight. The samples were heated at 70 °C for more than 9 h. The embedded samples were sectioned at 2-μm thickness using a rotary microtome (Leica) and stained or not stained (for GUS-stained tissues) by basic fuchsin before observations using a Nikon Eclipse 80i microscope equipped with a Nikon C-C phase contrast turret condenser (Nikon).

### Paraffin sectioning

For paraffin sectioning, the scutella of Nipponbare seeds cultured on N6 medium or CIM were fixed in ethanol-acetic acid (3:1, v/v), dehydrated through an ethanol-xylene series, stained with safranin, and embedded in paraffin. Then, 10-μm sections were cut with a microtome, mounted on slides covered with gelatin, deparaffinized in xylene, and rehydrated through an ethanol series before observations using a Nikon Eclipse 80i microscope equipped with a Nikon C-C phase contrast turret condenser (Nikon).

### Scanning electron microscope (SEM)

Plant tissues were fixed overnight at 4 °C in 2.5% glutaraldehyde in 0.1 M phosphate buffer (pH 7.0). Then the samples were prepared for SEM according to the previous method^31^ in Bio-ultrastructure Analysis Lab of Analysis Centre of Agrobiology and Environmental Sciences, Zhejiang University.

### RNA-seq analysis

For scutella collection, seeds of wild-type rice (Nipponbare) and *35S*_*pro*_*:MIR393b* were separately cultured on N6 medium (2 d, the control) and CIM (2, 5, 7 d). For each sample, scutella from 10 seeds were used to extract total RNA. Library construction and deep sequencing were carried out using the Illumina HiSeq 4000 platform (LC Bio, Hangzhou, China). The RNA-seq analysis (LC Bio, Hangzhou, China) was carried out using the reference genome in the Rice Annotation Project (RAP) database (https://rapdb.dna.affrc.go.jp/index. html) by the HISAT package v2.0^66^. Sequence-dependent bias and amplification noise were removed using UMI-tools v1.0.0^67^. The mapped reads for each sample were assembled using StringTie v1.3.4^68^. The differentially expressed mRNAs and genes were selected with the following criteria: log2 (fold change) >1 or log2 (fold change) < −1, and with statistical significance (*p* < 0.05) using the R package – edgeR^69^.

WGCNA analysis were performed as previously described^30^. The parameters of WGCNA program were as follows: gene expression > 1; soft threshold = 1 (estimate value); deep split = 2; min module size = 30; merge cut height = 0.1. In each module, the genes with eigengene-based connectivity value (|KME|) > 0.9 and topological overlap measure (TOM) value > 0.25 were regarded as hub genes.

The list of transcription factors in *Oryza sativa subsp. japonica* was from Plant Transcription Factor Database (http://planttfdb.gao-lab.org/)^70^. The GO analysis and heat-map of differentially expressed genes normalized by Z-score was constructed using tools in Omicstudio (https://www.omicstudio.cn/login).

Transcriptome sequencing data of the rice embryo (GSE179838)^31^, the rice leaf (GSE157400)^32^, the rice root (GSE217725)^33^, the rice shoot (GSE217725)^33^, and vasculature-derived callus from rice leaf explants (GSE86869)^7^ were published previously. The sequencing data were filtered using fastp v0.23.2^71^ with default parameters and aligned to the *Oryza sativa* genome (IRGSP-1.0) using hisat2 v2.2.1^72^, and gene expression was quantified using featurecounts v2.0.1^72^. To identify tissue/organ-specific expression genes, the mean TPM values among different replicates for each tissue/organ were used, and the maximum mean TPM value was selected as the representative for multiple time points. A specificity measure (SPM)^73^ threshold of 0.90 was applied to determine tissue/organ-specific expression genes. We identified 767 embryo-specific genes, 474 leaf-specific genes, 616 root-specific genes, and 133 shoot-specific genes. In the transcriptome data of vasculature- or scutellum-derived callus, the genes that were upregulated during the induction process on CIM at a 1.5-fold change threshold were collected. For the transcriptome with replicates, differential gene expression was determined using Deseq2 v1.36.0^74^ with an adjusted p-value threshold of <0.05; for the transcriptome without replicates, fold change was calculated based on TPM values. The proportion of upregulated genes among tissue/organ-specific expression genes was calculated for both transcriptomes and visualized using a heatmap generated by pheatmap v1.0.12 (Kolde R, 2019. pheatmap: Pretty Heatmaps. R package version 1.0.12, https://CRAN.R-project.org/package=pheatmap).

### RT-PCR and qRT-PCR

RT-PCR and qRT-PCR were performed as previously described^11,61^. *UBIQUITIN5* (*OsUBQ5*) served as the control. The primers used for PCR are listed in Supplementary Table 1.

### Statistics and reproducibility

Two-sided Student’s t-test was used in this study for statistical analysis. For callus weight analysis, four (Fig. 2d) or nine (Fig. 5g) biological replicates were performed. For qRT-PCR analysis, three biological replicates were performed. Details of statistics and reproducibility are described in figure legends or method.

### Reporting summary

Further information on research design is available in the Nature Portfolio Reporting Summary linked to this article.

## Supplementary information

Supplementary information

Description of Additional Supplementary Data

Supplementary Data 1

Supplementary Data 2

Reporting Summary

## Data Availability

All data and genetic materials used in this study are available from the corresponding authors upon request. The RNA-seq data obtained in this study have been deposited in the Gene Expression Omnibus (http://www.ncbi.nlm.nih.gov/geo/) under the accession number GSE179594, and can be accessed at http://xulinlab.cemps.ac.cn/. Sequence data from this article can be found in the Rice Annotation Project (https://rapdb.dna.affrc.go.jp/index.html) under the following accession numbers: *OsTIR1* (*Os05g0150500*), *OsAFB2* (*Os04g0395600*), *OsIAA11* (*Os03g0633500*), *OsCRL1* (*Os03g0149000*), *OsPIN1a* (*Os06g0232300*), *OsPIN1c* (*Os11g0137000*), *OsPIN1d* (*Os12g0133800*), *OsYUCCA1* (*Os01g0645400*), *OsIAA4* (*Os01g0286900*), *OsIAA5* (*Os01g0675700*), *OsIAA26* (*Os09g0527700*), *OsARF4* (*Os01g0927600*), *OsARF5* (*Os02g0141100*), *OsARF8* (*Os02g0628600*), *OsARF12* (*Os04g0671900*), *OsARF13* (*Os04g0690600*), *OsARF16* (*Os06g0196700*), *OsARF19* (*Os06g0702600*), *OsARF23* (*Os11g0523800*), *OsWOX2* (*Os01g0840300*), *OsWOX9C* (*Os05g0564500*), *OsPLT1* (*Os04g0653600*), *OsPLT2* (*Os06g0657500*), *OsPLT5* (*Os01g0899800*), *OsPLT6* (*Os11g0295900*), *OsPLT7* (*Os03g0770700*), *OsPLT9* (*Os03g0232200*), *OsVP1* (*Os01g0911700*), *OsLEC1* (*Os02g0725700*), and *OsLFL1* (*Os01g0713600*). Uncropped gel images are available in Supplementary Fig. 9. Numerical source data in Figs. 2d, 4c, 5g, and Supplementary Fig. 8c are shown in Supplementary Data 2.
