# Supplementary material for: Initiation of scutellum-derived callus is regulated by an embryo-like developmental pathway in rice
Source: Commun Biol. 2023 Apr 25;6:457. doi: 10.1038/s42003-023-04835-w (PMC10130139; doi:10.1038/s42003-023-04835-w)
Supplement: Supplementary file 1 — Supplementary information [file 42003_2023_4835_MOESM1_ESM.pdf]

Supplementary information

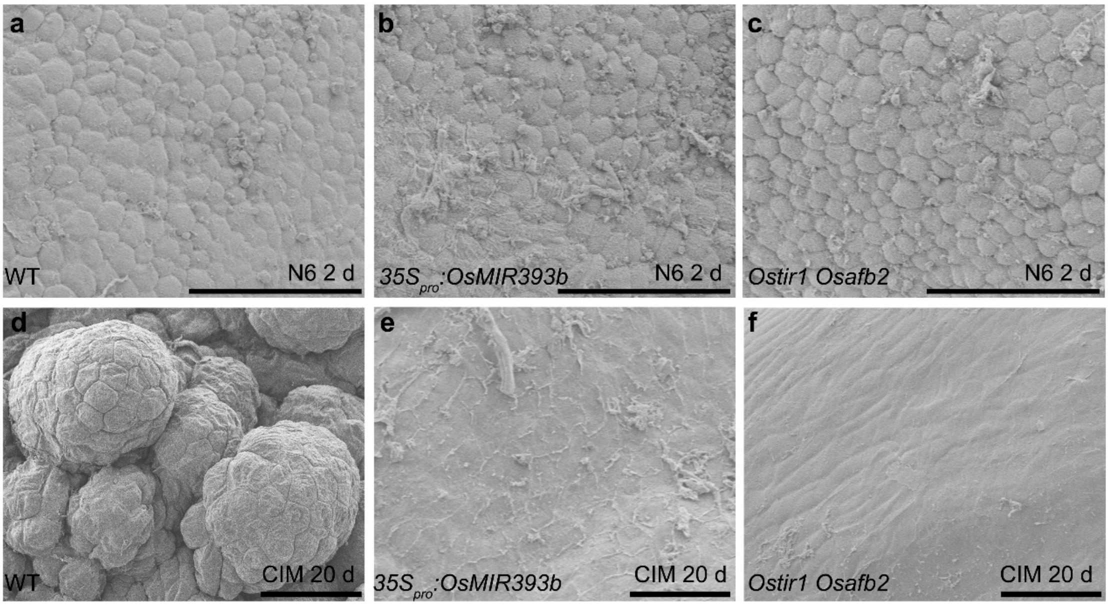

Supplementary Fig. 1 SEM analysis of scutellum-derived callus formation.

a–**f** SEM analysis showing scutellum of the wild type (**a, d**), *35S$_{pro}$:OsMIR393b* (**b, e**), or *Ostir1 Osafb2* (**c, f**) cultured on N6 medium without auxin for 2 days (the control, **a**–**c**) or on CIM for 20 days (**d**–**f**). Note that scutellum-derived callus formation could be observed from the wild-type scutellum (**d**) but not from the scutellum of *35S$_{pro}$:OsMIR393b* (**e**) or *Ostir1 Osafb2* (**f**) cultured on CIM.

Scale bars, 100 μm (**a**–**f**).

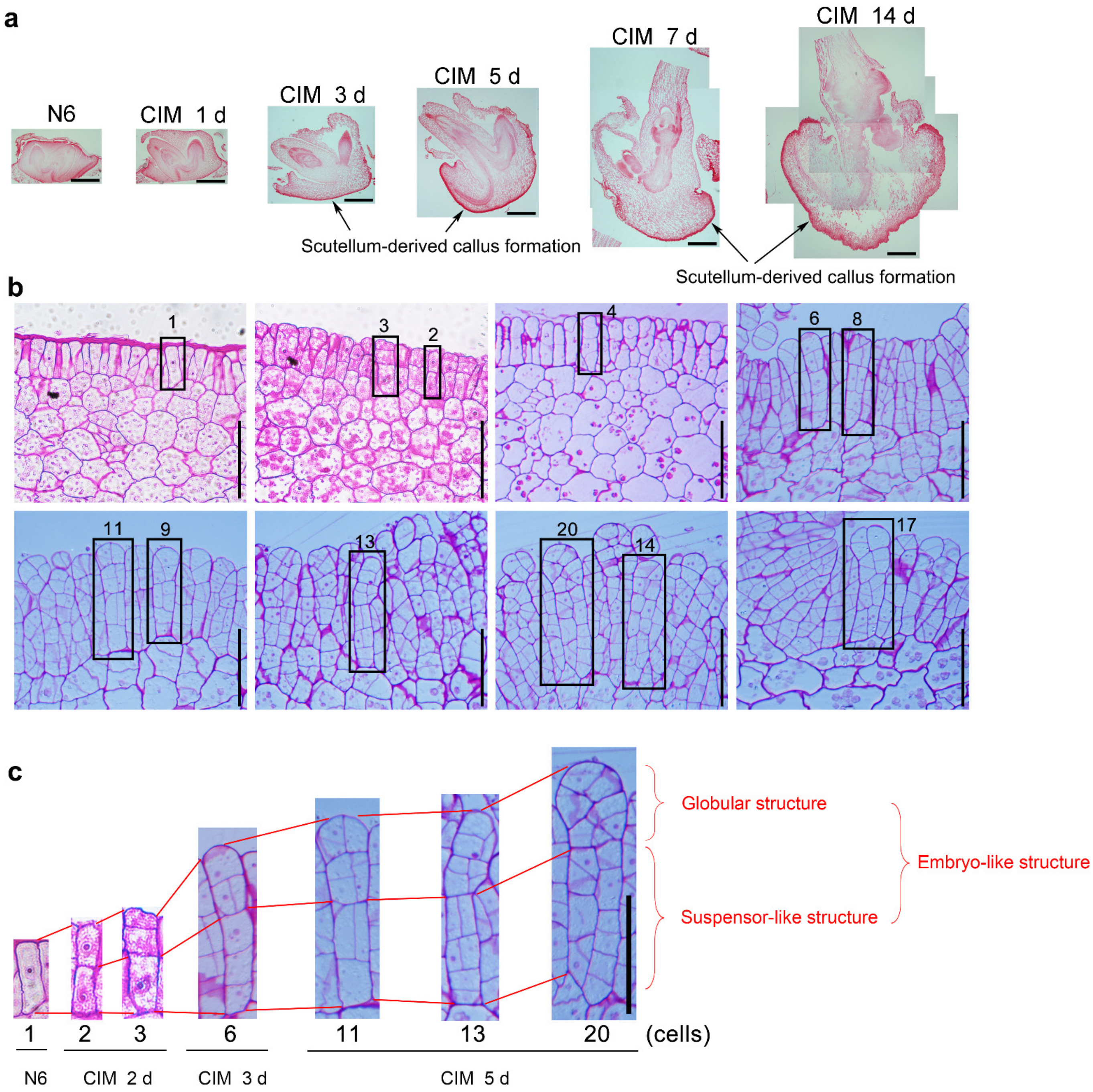

Supplementary Fig. 2 Sectioning analysis of scutellum-derived callus formation from wild-type scutellum.

a Time-lapse sections showing scutellum-derived callus formation from scutellum of wild-type rice seeds on CIM. Some sections were pasted together from small pictures of the same explant because the microscope was unable to capture the entire explant in a single visual field. Fig. 1e is the close-up view of the explant on CIM for 5 days in **(a)**. The explant on N6 medium for 1 day serves as the control.

b, c Time-lapse sections. The first-round cell division led asymmetric cell fates. The apical cell developed into the globular structure, and the basal cell developed into the suspensor-like structure. The globular structure could further develop into the scutellum-derived callus. Cells in (**c**) are close-up of some boxed regions in (**b**). Numbers indicate cell numbers during callus initiation. The explant on N6 medium for 1 d serves as the control.

Scale bar, 500 μm (**a**), 50 μm (**b, c**).

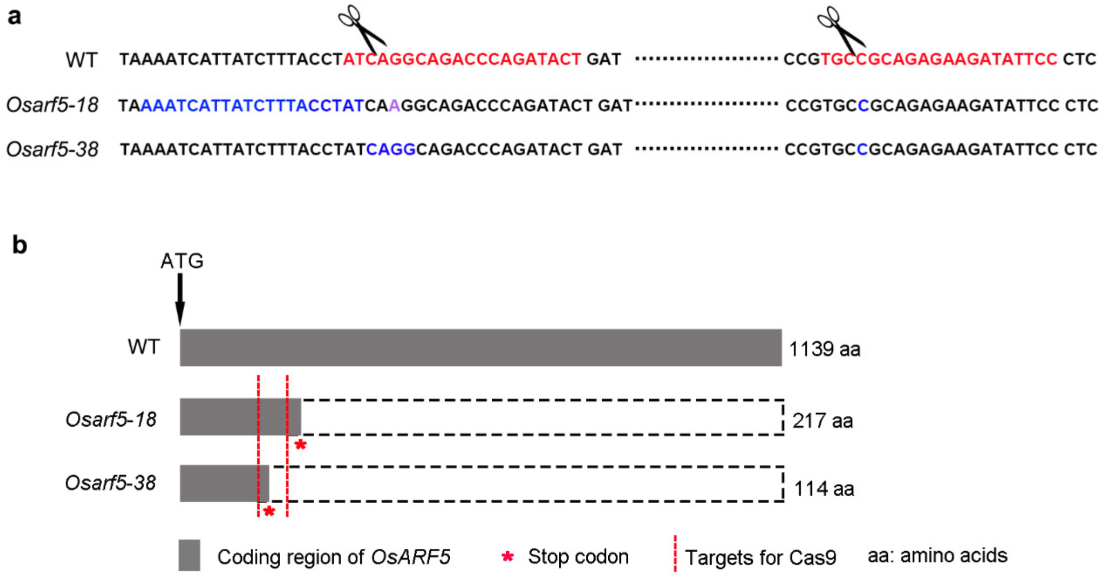

Supplementary Fig. 3 Generation of *Osarf5-18* and *Osarf5-38* mutants by CRISPR/Cas9.

a Genomic information of *Osarf5-18* and *Osarf5-38* mutants. Red letters indicate sequences of guide RNAs; blue letters indicate sequence deletion; the purple letter indicates sequence insertion.

b Proteins of *Osarf5-18* and *Osarf5-38* mutants.

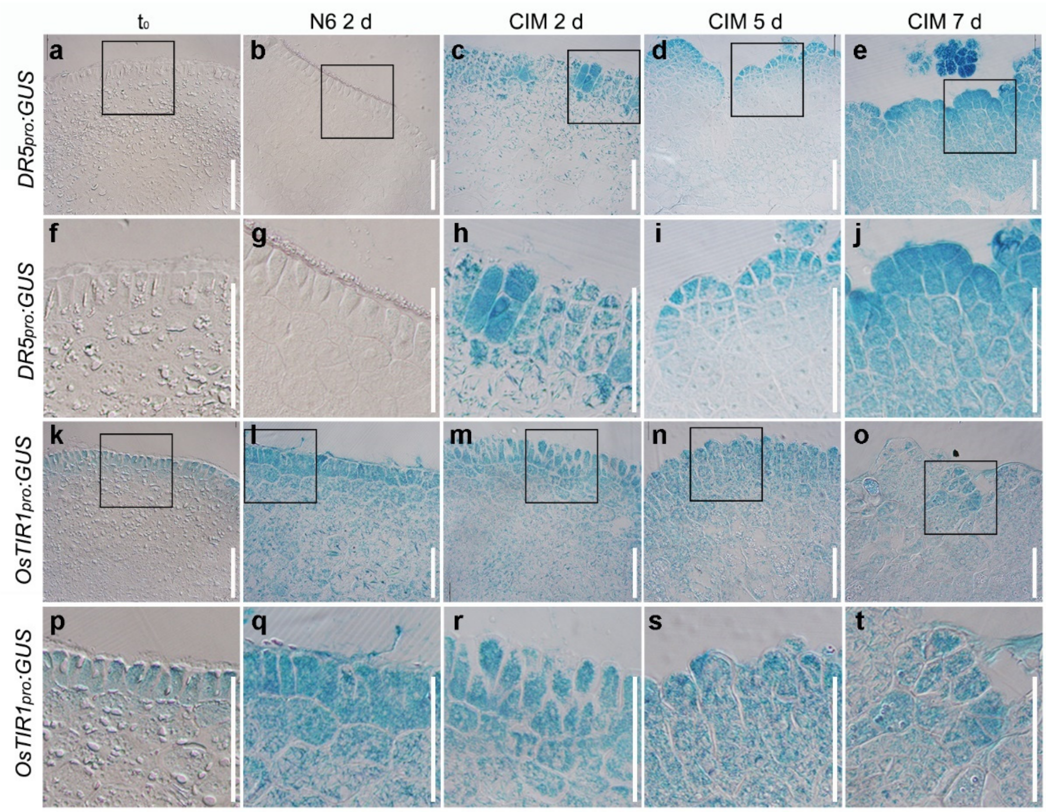

Supplementary Fig. 4 Auxin markers in rice scutellum-derived callus formation.

a–t GUS staining of *DR5*$_{pro}$*:GUS* (**a–j**) and *OsTIR1*$_{pro}$*:GUS* (**k–t**) during callus formation from scutellum on CIM or N6 medium (control). **f–j** and **p–t** are close-ups of the boxed regions in **a–e** and **k–o**, respectively.

Scale bars, 50 µm (**a–t**).

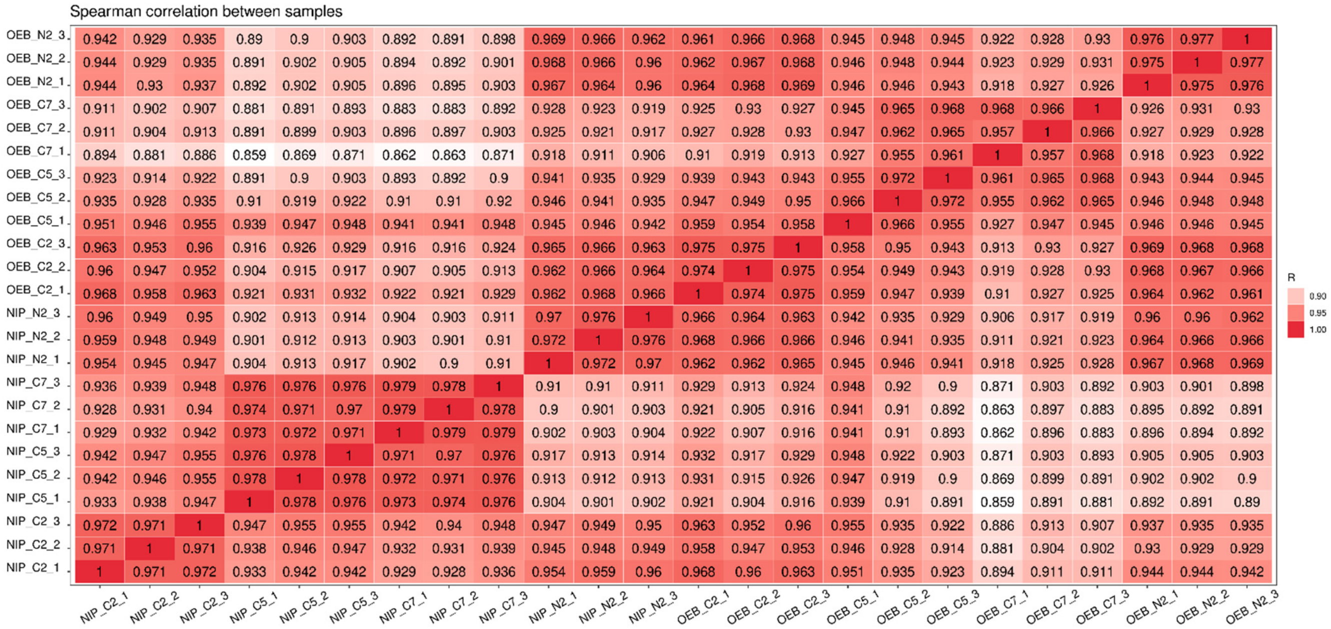

Supplementary Fig. 5 Quality control of the RNA-seq data.

Spearman's correlation test was used to evaluate the correlation of the three biological replicates in RNA-Seq. OEB, *$35S_{pro}$:MIR393b*; NIP, Nipponbare; N2, N6 medium for 2 days; C2, CIM for 2 days; C5, CIM for 5 days; C7, CIM for 7 days.

a

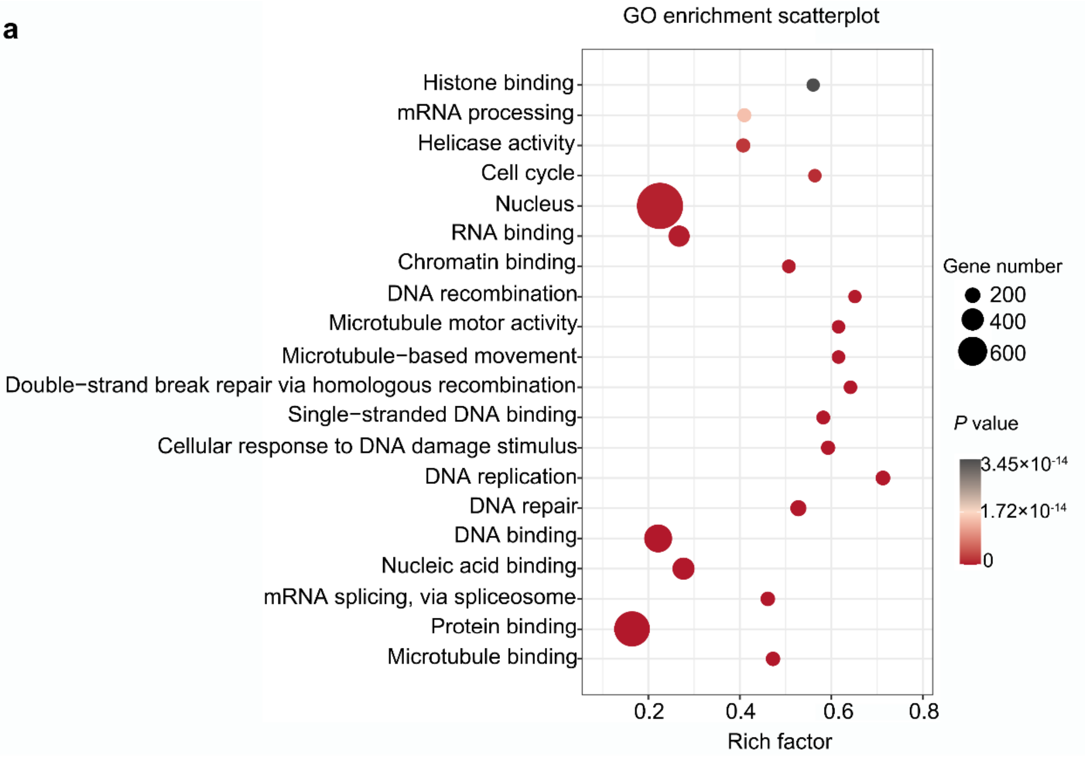

b

GO ID	GO Term	S gene number	*P* value
GO:0009793	embryo development ending in seed dormancy	23	0.00
GO:0009553	embryo sac development	6	0.01
GO:0009558	embryo sac cellularization	3	0.01
GO:0009791	post-embryonic development	3	0.08
GO:0010086	embryonic root morphogenesis	1	0.12
GO:0010069	zygote asymmetric cytokinesis in embryo sac	1	0.12
GO:0045694	regulation of embryo sac egg cell differentiation	1	0.12
GO:0045995	regulation of embryonic development	1	0.22
GO:0009880	embryonic pattern specification	1	0.39
GO:0090698	post-embryonic plant morphogenesis	1	0.67

Supplementary Fig. 6 Gene Ontology (GO) analysis.

a Top 30% genes in the module turquoise were used for GO analysis. Top 20 GO terms by *P* values were shown.

b Some GO terms related to embryo and embryo development.

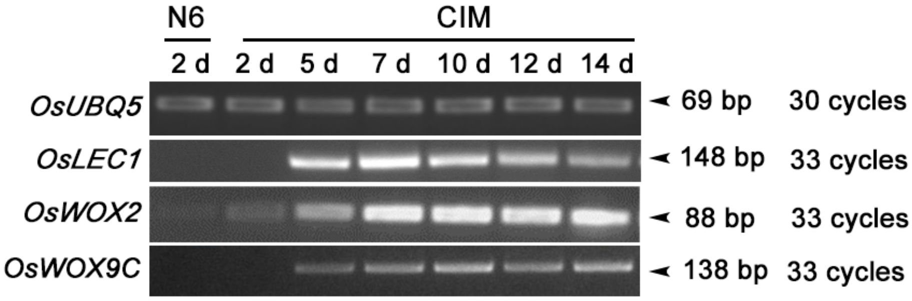

Supplementary Fig. 7 RT-PCR analysis of auxin-activated genes.

RT-PCR analyses of auxin-activated genes in wild-type seeds cultured on N6 medium or CIM, using RNA extracted from wild-type scutella. The bands of different genes are derived from different gels. The predicted band sizes were indicated. Two biological repeats were analyzed and showed the same result.

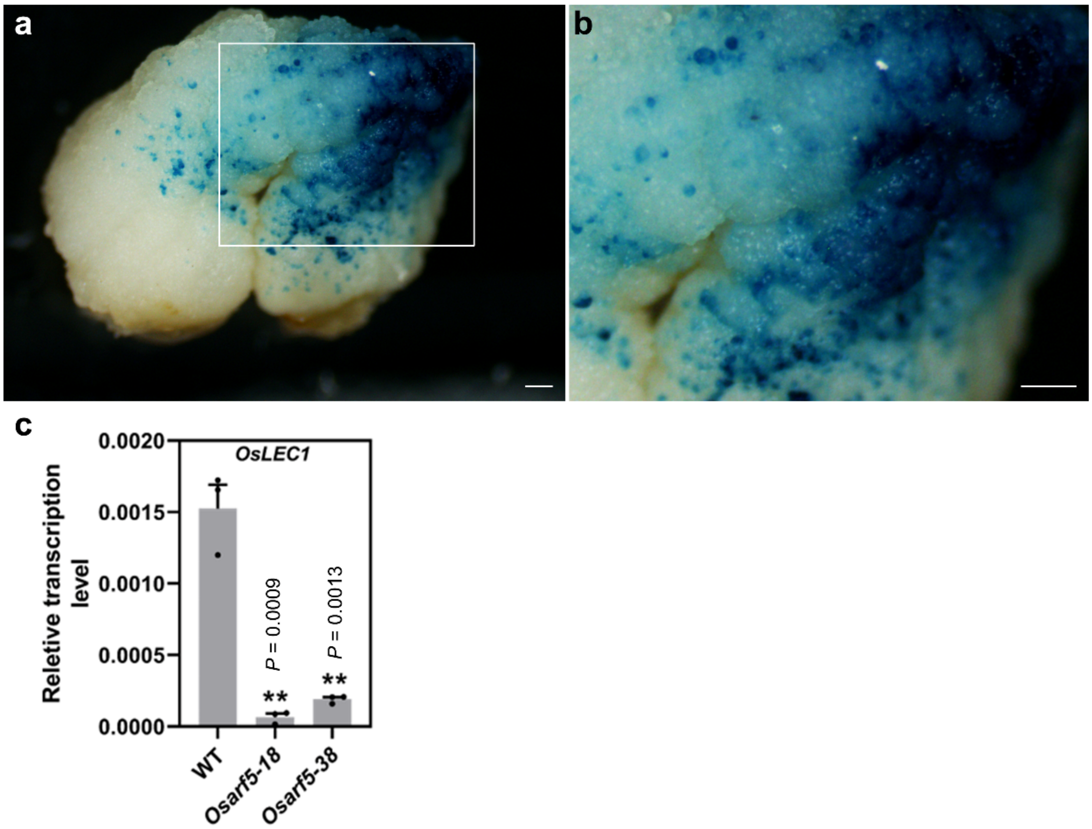

Supplementary Fig. 8 Analysis of *OsLEC1* in callus formation.

a, b GUS staining of the *OsLEC1*$_{pro}$*:GUS* seed cultured on CIM for 7 d, showing that the *OsLEC1* promoter was highly activated in scutellum-derived callus. **(b)** is the close-up view of the boxed region in **(a)**.

c qRT-PCR analysis of the *OsLEC1* expression level in calli from wild type and *Osarf5* mutants. The mature seeds were cultured on CIM for 7 days and then scutella were collected for RNA extraction. The data are presented as mean values ± s.e.m. from three biological replicates (three technical replicates in each biological replicate). The individual values are indicated by dots. $**P < 0.01$ in a two-sided Student's t-test compared with the wild-type control.

Scale bars, 500 μm **(a, b)**.

Supplementary Fig. 9 Uncropped gel images.

The orange boxes indicate the cropped gel images used in figures.

Uncropped images of RT-PCR in Fig. 5k.

OsUBQ5

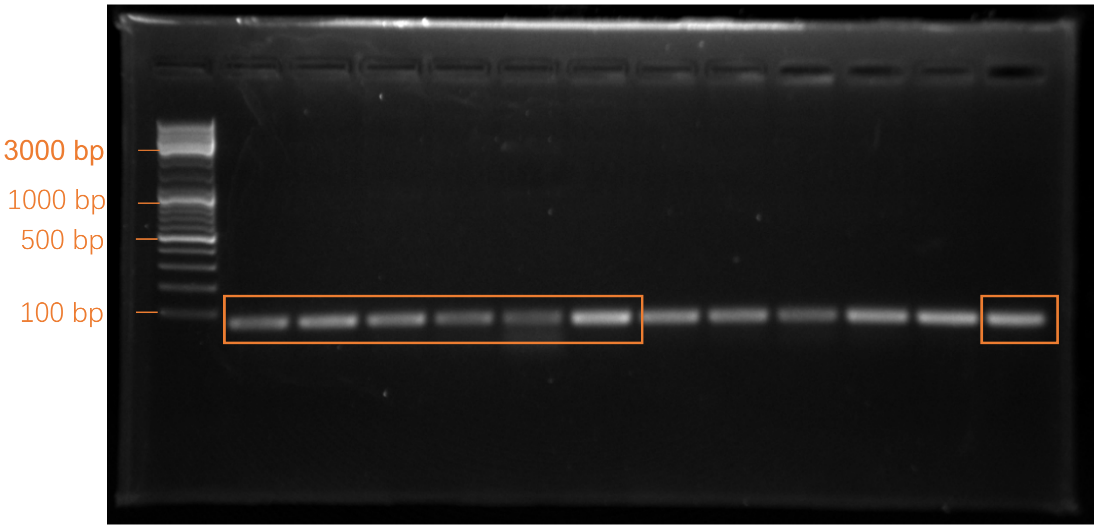

OsLEC1

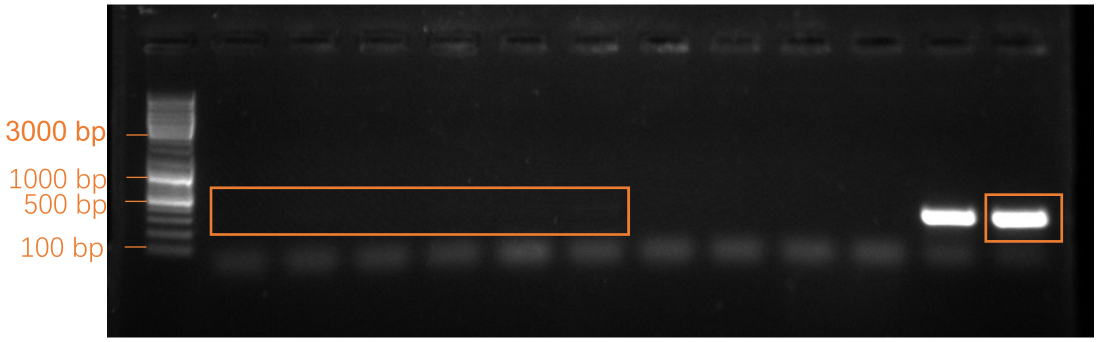

Uncropped images of RT-PCR in Supplementary Fig. 7.

OsUBQ5

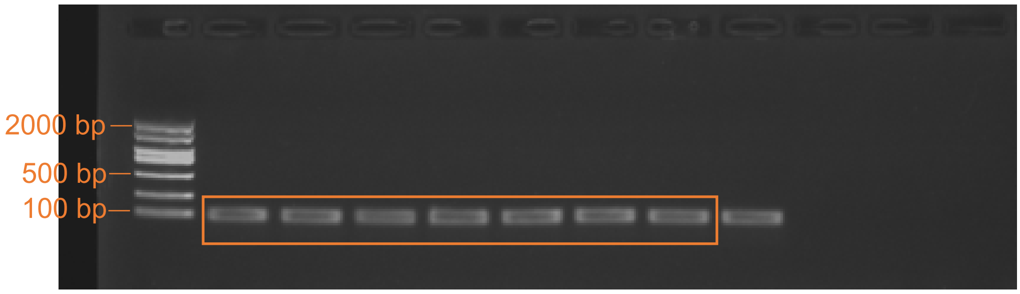

OsLEC1

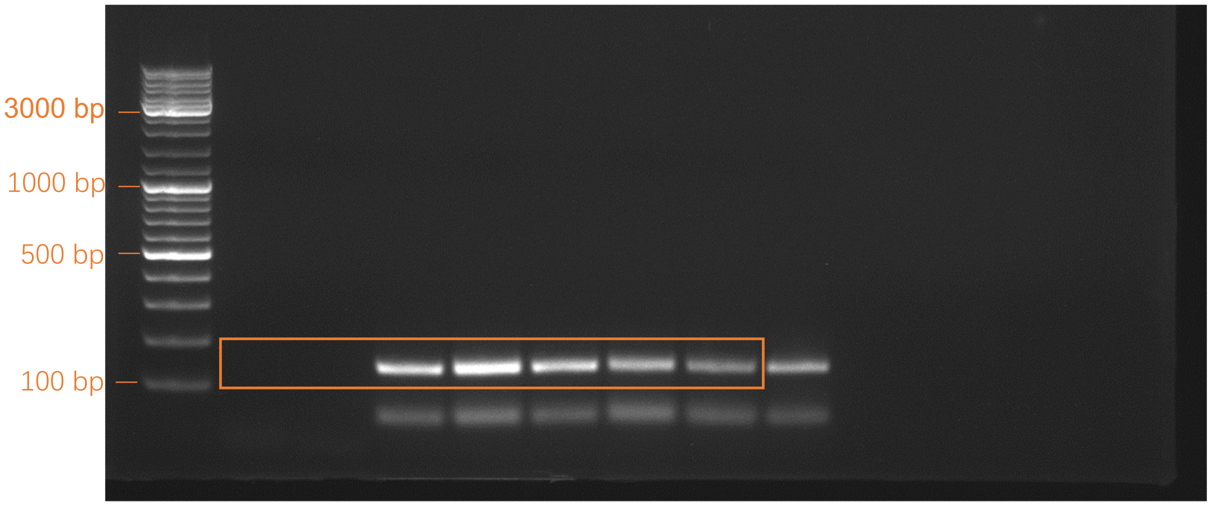

OsWOX2

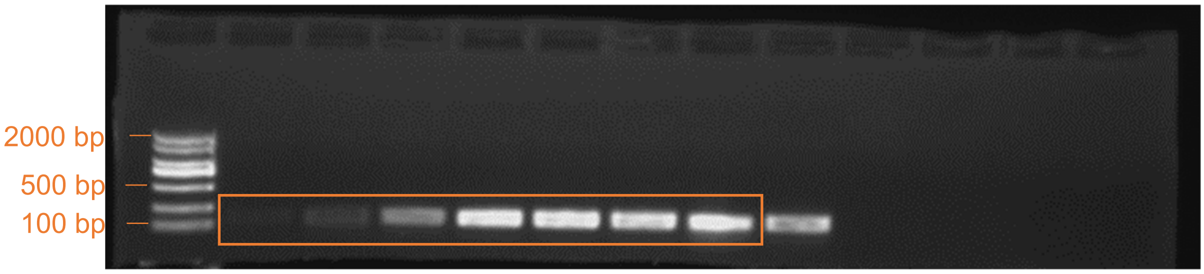

OsWOX9C

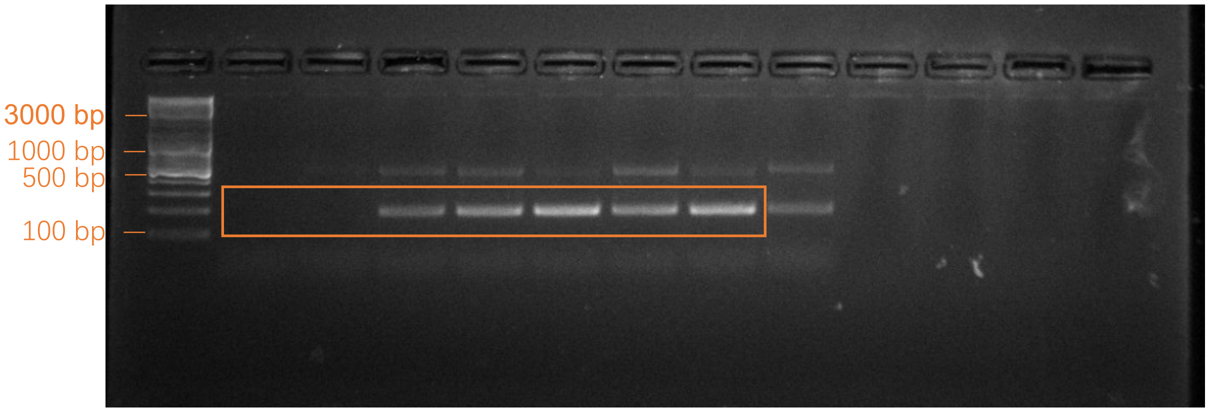

Supplementary Table 1 List of primers used in this study.

Primers for RT-PCR	Sequences (5'- 3')
OsLEC1 RT F	CGTCGGTGGGATGCTCAAGTC
OsLEC1 RT R	GGTGCTCGAAGTTGACGGTCT
OsWOX2 RT F	GAACCCGTCGTGCTTTTTGAA
OsWOX2 RT R	AGCAGTACACCTTCCAATCCC
OsWOX9C RT F	CCATCGACGATCTCACCCAG
OsWOX9C RT R	GTCGTCCATGGAATGGTGGT
OsLEC1 QRT/RT F	GGCTATACGTGAGCAGGACC
OsLEC1 QRT/RT R	CCCGGTGATGAAGCTGATGT
OsUBQ5 QRT/RT F	ACCACTTCGACCGCCACTACT
OsUBQ5 QRT/RT R	ACGCCTAAGCCTGCTGGTT

Supplementary Data 1 RNA-seq and WGCNA analysis of rice callus formed from wild-type and *$35S_{pro}$:OsMIR393b* scutella on CIM.

Supplementary Data 2 Numerical source data.
