# Supplementary material for: Initiation of scutellum-derived callus is regulated by an embryo-like developmental pathway in rice
Source: Commun Biol. 2023 Apr 25;6:457. doi: 10.1038/s42003-023-04835-w (PMC10130139; doi:10.1038/s42003-023-04835-w)
Supplement: Supplementary file 2 — Description of Additional Supplementary Data [file 42003_2023_4835_MOESM2_ESM.docx]

**Description of Additional Supplementary Files**

**File name:** Supplementary Data 1

**Description:** RNA-seq and WGCNA analysis of rice callus formed from wild-type and 35Spro:OsMIR393b scutella on CIM.

**File name:** Supplementary Data 2

**Description:** Numerical source data.
